# A Refined Nomenclature System to Better Discriminate Endo- and Exo-Type Fructanases and Glucanases

**DOI:** 10.3390/biom15010011

**Published:** 2024-12-25

**Authors:** Laura Leaerts, Jaime Ricardo Porras-Domínguez, Maxime Versluys, Wim Van den Ende

**Affiliations:** Laboratory of Molecular Plant Biology and KU Leuven Plant Institute, Kasteelpark Arenberg 31, 3001 Leuven, Belgium; laura.leaerts@kuleuven.be (L.L.); jaimericardo.porrasdominguez@kuleuven.be (J.R.P.-D.); maxime.versluys1@gmail.com (M.V.)

**Keywords:** endo/exo mechanism, fructanases, glucanases, GH32 family, inulin, levan, nomenclature

## Abstract

Distinguishing between endo- and exo-type enzymes within the glycoside hydrolase (GH) classification presents significant challenges. Traditional methods, often based on endpoint activity measurements, do not capture the full range of products generated, leading to inconsistencies in classification. Not all exo-acting fructanases and glucanases produce monosaccharides (like fructose or glucose), while endo-acting enzymes do not solely produce higher-degree polymerization oligosaccharides. In practice, both enzyme types can yield a variety of products throughout the reaction, complicating classification efforts. To address these challenges, we propose a refined nomenclature system for GH enzymes, including fructanases and glucanases, based on good practices and initial product formation. This system classifies enzymes into four categories for each type: Fr, Fn, Fn,n+1 and F1 for fructanases, and Gr, Gn, Gn,n+1 and G1 for glucanases, based on their mode of action (endo- or exo-based) and initial product profiles. Our refined nomenclature system will advance enzyme structure–function research and support the production and application of fructan and glucan oligosaccharides as prebiotics, priming agents, and potential signaling molecules in microbe–microbe and plant–microbe interactions. Ultimately, this system could benefit agronomy and the food industry, contributing to health improvements.

## 1. Introduction

**Figure 1:**
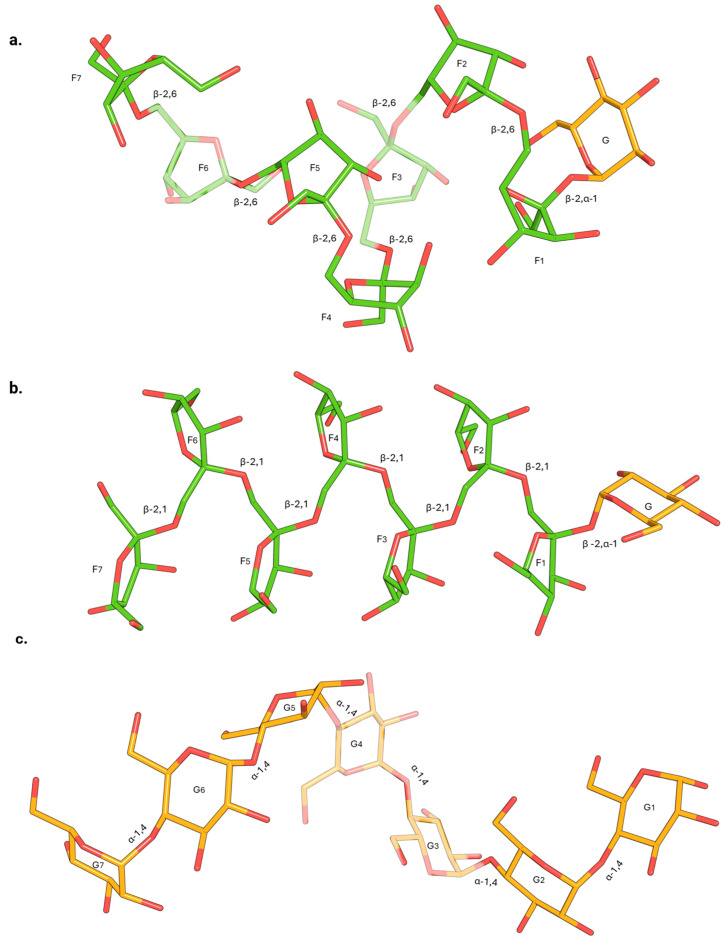
Chemical structures of selected oligosaccharides. Panel (**a**) illustrates the chemical structure of a linear non-reducing GF_7_-levan-type oligosaccharide composed of β-2,6 linkages. The terminal Glc residue is highlighted in orange, while the Fru units are distinguished in green. Panel (**b**) shows the chemical structure of a linear non-reducing GF_7_-inulin-type oligosaccharide consisting of β-2,1 linkages. Similarly, the terminal Glc residue is orange, while the Fru units are depicted in green. Naturally occurring reducing Fn-type fructans, lacking the terminal Glc residue, are also commonly found in nature. Panel (**c**) presents the chemical structure of a linear reducing G_6_ malto-oligosaccharide composed of α-1,4 linkages. G_1_ represents the reducing end, characterized by a free anomeric carbon. G = glucose; F = fructose. The figure was generated using PyMOL with the Glycam plugin.

Carbohydrates are among the most abundant compounds in nature and play essential roles in various biological functions [1]. Within this broad category, fructans and glucans are particularly significant due to their roles in energy storage and structural functions across different organisms [2,3]. The prebiotic properties of fructo- and malto-oligosaccharides (FOS and MOS) are also well documented [4,5]. Fructans are oligo- and polysaccharides primarily composed of fructose (Fru) units, while glucans are composed of glucose (Glc) units (Figure 1) [6,7]. Their biosynthesis involves distinct biochemical pathways. In most cases, glucan synthesis in microbes and plants is facilitated by glycosyltransferases (GTs) [8] that use activated sugars, such as UDP-glucose (UDP-Glc), as donor substrates (Leloir-type enzymes). Conversely, microbial levansucrases, inulosucrases, and a few amylosucrases [9,10] can use sucrose (Suc), a non-reducing disaccharide, as donor and initial acceptor substrates, leading to the synthesis of (mostly linear) fructans (β-2,6 levan; β-2,1 inulin) and glucans (α-1,4 amylose) (Figure 1). For reasons of simplicity, we focus on these particular types of microbial enzymes in this paper.

**Figure 2:**
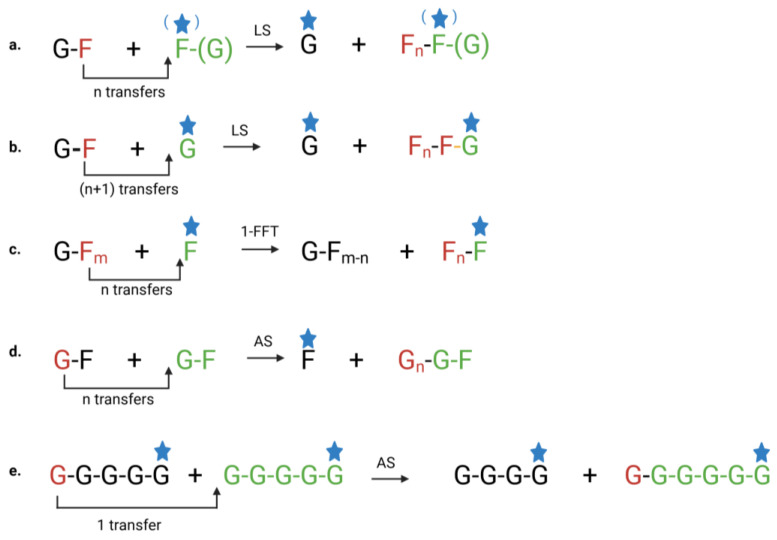
Biosynthesis of selected fructans and glucans. Panel (**a**) illustrates the levansucrase (LS)-mediated synthesis of bacterial levans of the GFn and Fn types. GFn-type levans, synthesized from a Suc donor and acceptor molecule, have a non-reducing sucrosyl unit at one end (highlighted in green), which cannot be targeted by exo-type GH32 fructanases. Instead, these enzymes act on the opposite non-reducing Fn end (highlighted in red), where terminal Fru residues are located. In contrast, Fn-type levans, synthesized from a Suc donor with Fru as an acceptor molecule, possess a reducing Fru end (denoted by the blue asterisk), which also resists attack by exo-type fructanases. Also, in this case, the terminal red-highlighted Fru unit serves as the target for the exo-type fructanase reaction. Panel (**b**) illustrates the progressive action of LS, where Suc serves as the donor substrate and Glc as the acceptor molecule, leading to the formation of blastose (Fruβ2,6Glc; linkage indicated in orange), a reducing sugar. This intermediate can then be elongated by adding (Fruβ2,6Fru) linkages through repeated Fru transfer, resulting in longer blasto-fructan chains. The reducing end of the formed product is marked with a blue asterisk, while the exo-type GH32 enzymatic reaction begins at the opposite non-reducing Fn end (indicated in red). Glc is produced as a byproduct. Panel (**c**) illustrates the synthesis of Fn-type fructans by plant 1-FFT enzymes. Using inulin-type fructans (with m > n) as donor substrates and Fru as an acceptor molecule, these enzymes produce Fn+1 and GFm-n oligosaccharides. The reducing end is marked with a blue asterisk, and breakdown by fructanases initiates from the non-reducing Fm-n (black) and Fn (red) ends. Panel (**d**) depicts the structural differences in bacterial FGn- and Gn-type glucans. Both biosynthesis processes are catalyzed by amylosucrase (AS). The process starts with the cleavage of the α-1,β-2 glycosidic bond of the Suc donor, followed by the transfer of a glucosyl group to an acceptor Suc. Repeated glucosyl transfers result in the formation of FGn oligosaccharides and the release of Fru. These bacterial FGn-type glucans feature a non-reducing sucrosyl unit at one end (illustrated in green). The terminal G residue (red) indicates the target site for exo-type amylase attack. Panel (**e**) illustrates the disproportionation reactions catalyzed by AS, involving MOS with a degree of polymerization (DP) higher than 4. This process results in the elongation of the acceptor glucan chain, matching the length of the donor substrate (here composed of 5 Glc moieties), while preserving its reducing end (indicated by the blue asterisk). The red and black Glc units depict the target sites of exo-acting amylases. G = glucose; F = fructose; blue asterisk = reducing end; red end = target site of fructanases or glucanases. Figure created using BioRender.com, accessed on 15 November 2024.

When fructans are synthesized from Suc as a single substrate (also termed GFn-type fructans), or glucans are synthesized from Suc as the only substrate (FGn type), they do not have a true reducing end, characterized by a free anomeric carbon (Figure 1 and Figure 2).

The absence of a genuine reducing end complicates the terminology used to describe the subsequent mechanistic directionality of their degradation. It should be noted that truly reducing Fn-type fructans, lacking a terminal Glc, also occur in nature. They are biochemically less stable as compared to GFn-type fructans [11]. They can be directly produced through the transfer of fructosyl units to acceptor Fru (see Section 1.1) or by the action of microbial endo-type fructanases [6]. Plants lack endo-fructanases [12].

There is often confusion about whether fructan- and glucan-hydrolytic microbial enzymes function in an exo- or endo-type manner. Here, we propose an alternative nomenclature that better distinguishes between endo- and exo-type fructanases and glucanases. By extension, these concepts could also be applied to other types of GH enzymes.

### 1.1. Enzymatic Mechanism of Levansucrases in Bacterial Levan Biosynthesis

In microbes, two main groups of fructansucrases are responsible for the biosynthesis of levan and inulin: levansucrase (EC 2.4.1.10) and inulosucrase (EC 2.4.1.9) [13]. These enzymes belong to GH68. In this biosynthetic pathway, Suc acts as fructosyl donor. They facilitate the transfer of Fru units from the donor Suc, leading to the progressive elongation of the growing fructan chain with one fructosyl unit at a time [13]. The degree of polymerization (DP) reached in microbes is usually much higher than observed in plant fructans [13].

As illustrated in Figure 2a, bacterial GFn-type levans, synthesized from Suc as a single substrate, possess a non-reducing sucrosyl unit at one end. Exo-type fructanases cannot attack this end, since GH32 fructanases cannot process the terminal Glc [14]. Instead, GH32 fructanases attack the opposite non-reducing end where terminal Fru residues reside (Figure 2a). In case Fru is used as an acceptor substrate, Fn-type levans with a reducing end can be formed (Figure 2a) [15,16]. Exo-type fructanases can also not attack this reducing end [14].

During the progressive action of some levansucrases, the accumulating Glc can also be used as an acceptor to create blastose (Fruβ2,6Glc), a reducing sugar [15,16], which can be further elongated through (Fruβ2,6Fru) linkages (Figure 2b) [16].

Notably, plant fructan/fructan 1-fructosyl transferase (1-FFT) enzymes can also synthesize Fn-type fructans (Figure 2c). However, inulin-type fructans are used as a donor substrate. The produced Fn oligosaccharides contain Fruβ2,1Fru linkages, and they are termed inulo-oligosaccharides (IOS) [12].

### 1.2. The Role of Bacterial Amylosucrase in Linear α(1,4)-Glucan Formation

Among α-glucan-synthesizing enzymes, one type of bacterial amylosucrase is notable for its ability to use Suc, instead of UDP-Glc, as a donor substrate. Suc can also be used as an acceptor substrate [9]. This enzyme, classified as GH13, was initially discovered in *Neisseria perflava* [17], and its reaction mechanisms were later studied in *Neisseria polysaccharea* [9,10].

The biosynthesis of bacterial linear α-1,4 glucans (amylose), as catalyzed by amylosucrase (EC 2.4.1.4), is illustrated in Figure 2d. This process begins with the cleavage of the α-1,β-2 glycosidic bond of the Suc donor. Subsequently, the glucosyl group is transferred to the Glc moiety of an acceptor Suc. Repeated transfers of glucosyl groups result in the formation of FGn-type oligosaccharides and the release of free Fru [9]. Exo-type glucanases cannot attack the non-reducing sucrosyl end, since they cannot process the terminal Fru. Instead, they attack the opposite non-reducing end where terminal Glc residues reside (Figure 2d).

It seems possible that the terminal Fru of FGn-type oligosaccharides is trimmed by exo-type GH32 fructosidase enzymes, but this requires experimental verification. In this way, reducing Gn-type MOS could be obtained, which can be further used by disproportionation reactions between MOS > DP4 (Figure 2e) [10].

This process results in the elongation of the acceptor glucan chain, retaining its reducing end. It was proposed that the intrinsic disproportion reactions may be the most prominent ones *in vivo*. However, when MOS > DP4 is absent at initial stages, Suc may be crucial to initiate the formation of low-DP FGn, until it reaches a DP > 4 [9,10].

### 1.3. Mechanisms of Action for Exo- and Endo-GHs

Both fructanases (GH32) [6,14] and glucanases (various GH families) [18] can be classified as either exo- or endo-acting enzymes based on their specific cleavage sites along the fructan or glucan chain. Exo-type enzymes cleave terminal glycosidic bonds to progressively release single monosaccharides or small oligosaccharides from the chain. In contrast, endo-type enzymes typically target internal glycosidic bonds within the polysaccharide chains, resulting in a polydisperse mixture of reducing and non-reducing products [18,19,20,21,22]. The reducing products of levanases are termed levan oligosaccharides (LOS).

## 2. Problem Statement

**Figure 3:**
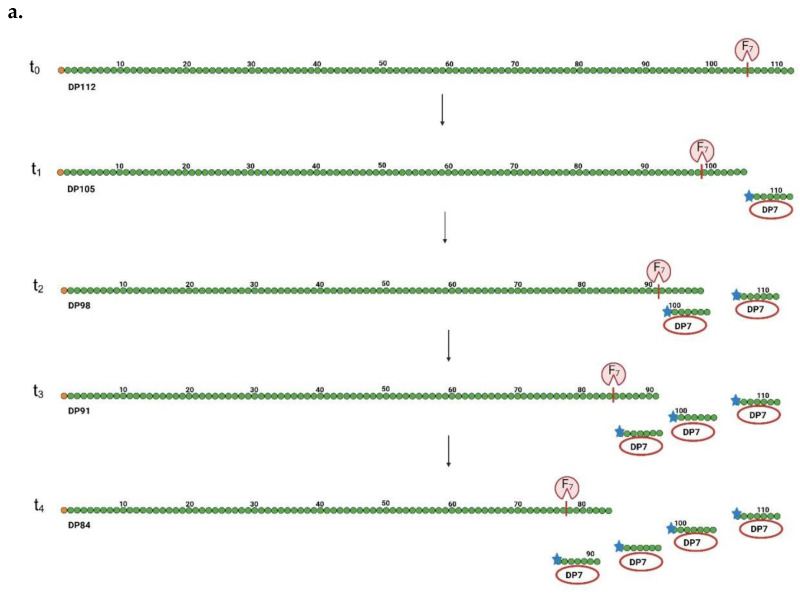

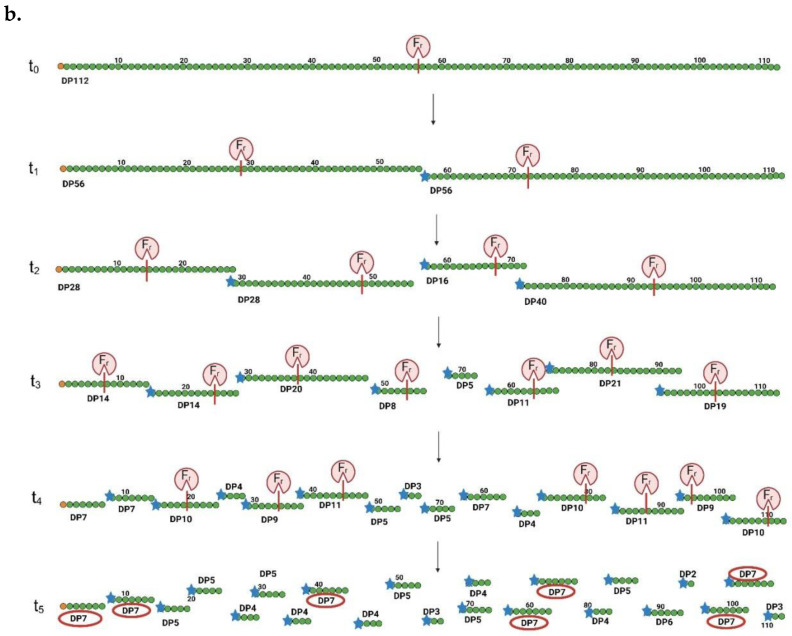
Principle of endo- and exo-acting fructanase activity. Panel (**a**): This panel illustrates the principle of time-dependent exo-acting fructanase activity, focusing on an enzyme that produces F7. According to our novel nomenclature system, this F7 enzyme produces Fn-type fructans with a degree of polymerization (DP) of 7. The panel emphasizes the critical importance of taking initial time measurements to accurately distinguish between endo- and exo-acting enzymes. Panel (**b**): This panel demonstrates the principle of Fr (endo) activity, which results in a polydisperse mixture of products with a dominant DP of 7. As in panel (**a**), initial time measurements are highlighted as essential for precisely characterizing the enzyme’s mode of action. Each product is labeled with its corresponding DP, demonstrating the enzymatic activity over time. In this context, “Fr” refers to the new terminology in our proposed nomenclature system, where ’r’ stands for random cleavage, underscoring the enzyme’s endo-acting nature. In both panels, the enzyme’s cleavage site is marked by a red line, while the reducing end is indicated by a blue asterisk. Green dots represent fructose units, while orange dots indicate glucose units. Red ovals signify F7 formation. Figure generated using BioRender.com, assessed 16 November 2024.

In the field of GH enzyme classification, distinguishing between endo- and exo-type enzymes poses several key challenges:

1. Enzymatic activity measurements: Current methods for classifying enzymes as endo- or exo-type often rely heavily on endpoint activity measurements, while other authors rely on initial product profiles. This can lead to pertinent inconsistencies and confusion. Endpoint data alone do not usually fully capture the enzyme’s overall activity profile. To accurately determine whether an enzyme is endo- or exo-acting, it is essential to focus on product formation at the very initial stages. However, product profiles at the intermediate and endpoint stages of the reaction may also be informative, but less critical for enzyme classification. All the generated product profiles should be carefully compared to a control (zero hour sample).
2. Product formation misconceptions: A common misconception is that exo-acting fructanases produce only Fru as an end product, while endo-acting fructanases exclusively produce a dispersed mixture of LOS or IOS. However, studies have shown that some exo-fructanases can produce products with DPs of two, three, or even up to eight but presumably with a more narrow product profile [23,24,25,26]. The other way around, besides LOS/IOS production, some endo-acting fructanases may also produce disaccharides (levanbiose or inulobiose) and some Fru, especially at their terminal stages [21,27]. This once again underscores the importance of considering initial product profiles, preferentially through advanced separation techniques (e.g., through High-Performance Anion Exchange Chromatography with Integrated Pulsed Amperometric Detection: HPAEC-IPAD).
3. Ambiguous classification because of technical limitations and differential interpretations: To better illustrate the confusion between endo- and exo-type enzyme products, we refer to two publications that report on DP7 and DP8 LOSs [24,26]. Some authors refer to these enzymes as endo-acting [28], while others categorize them as exo-acting [29]. A closer inspection of the data of [24] shows that Thin-Layer Chromatography (TLC) data were not shown, while their High-Pressure Liquid Chromatography (HPLC) analysis [24] showed a putative “DP7 peak”.
4. However, it is well known that, above a certain DP, higher DPs elute together as a mixture. Therefore, the “DP7 peak” may consist of a dispersed mixture of levans. Figure 3 compares the product spectra generated by exo- and endo-mechanism product formation, starting with DP112 levan. Clearly, differential product profiles are generated that can be discriminated by TLC or HPAEC-IPAD analysis as a function of reaction time. However, there is a complication because some enzymes may not consistently produce a defined DP (Figure 3) but rather a mixture of two (or perhaps even three) different DPs [30]. This further increases the chances of confusion between endo- and exo-type mechanisms. These can only be resolved by focusing on initial reaction times, careful comparison with zero hour control measurements, and the use of purified substrates with a sufficiently high DP, free from lower-DP sugar contaminants.

A similar confusion can occur with glucanases, where maltohexaose can be produced by both endo- and exo-type α-amylases [31,32]. Despite their different mechanisms, both enzymes may ultimately produce very similar end products, which can be misleading.

**Figure 4:**
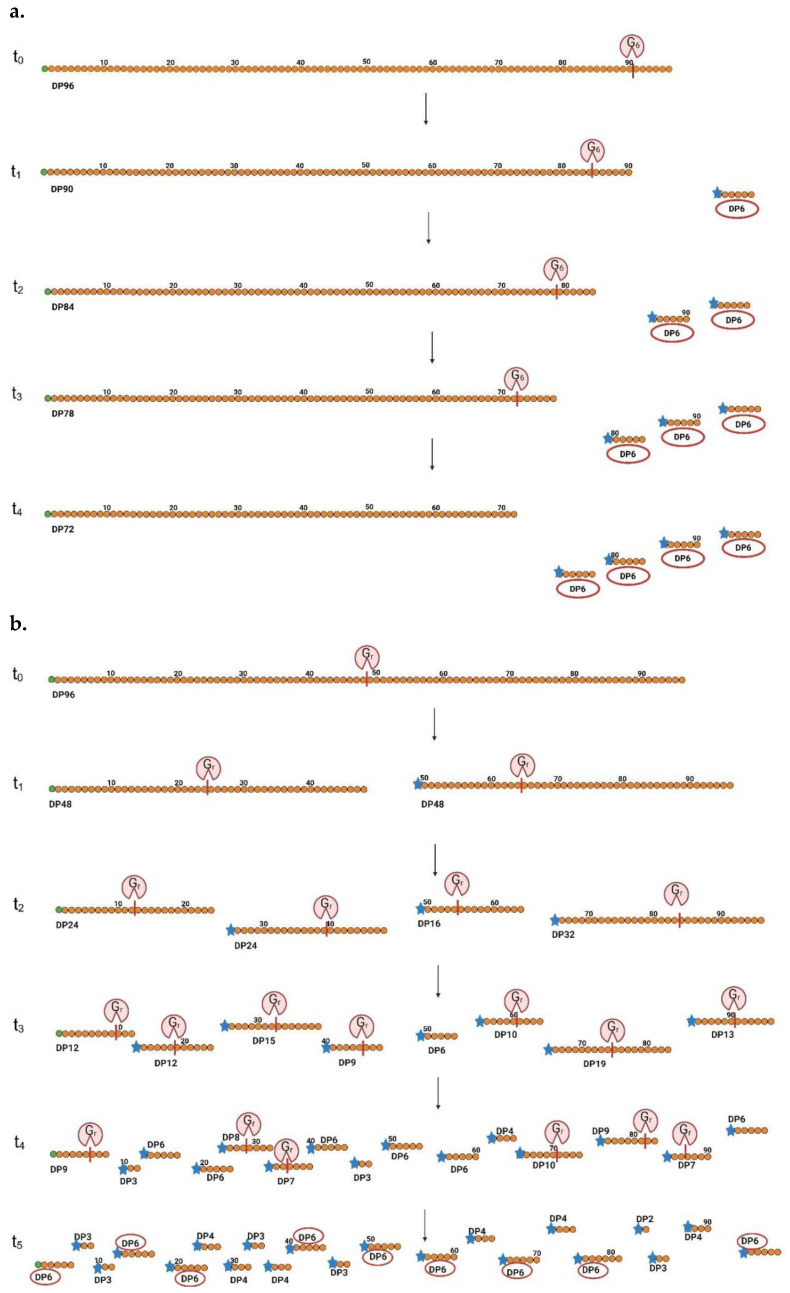
Principle of endo- and exo-glucanase activity. Panel (**a**): Time-dependent exo-type glucanase activity is depicted, resulting in the production of maltohexaose with a degree of polymerization (DP) of 6. In this panel, green dots represent Fru units, while orange dots denote Glc units. According to our new nomenclature system, G6 refers to a glucanase that produces DP6 products. The enzyme’s cleavage site is marked by a red line, and the blue asterisk indicates the reducing end. Panel (**b**): Time-dependent endo-type glucanase activity, leading to a polydisperse product profile, but with DP6 as a dominant product. In both panels, the enzyme’s cleavage site is marked by a red line, while the reducing end is indicated by a blue asterisk. Green dots represent fructose units, while orange dots indicate glucose units. Red ovals signify G6 formation. This figure was created using BioRender.com, assessed on 16 November 2024.

Figure 4 provides a schematic representation of these processes. This once again highlights the critical importance of measuring enzymatic activity and capturing product profiles at the very beginning of the reaction.

## 3. A New Classification System Discriminating Fr/Gr, Fn/Gn, Fn,n+1/Gn,n+1, and F1/G1 Enzymes

To address the current problems, there is an imperative need to refine the nomenclature system for exo- and endo-GH enzymes. This refinement seeks to more accurately represent enzyme product profiles derived from initial reaction times, while incorporating universally applicable “good practices” (see next section) to validate such classification. The goal is to establish a standardized approach, minimizing confusion and ensuring consistency in future research.

Our proposed fructanase nomenclature system includes four classes:

- **Fr**: Refers to random fructanases (endo-based), which do not produce significant amounts of Fru or fructan disaccharides at initial stages. Instead, they generate a very broad DP spectrum at intermediate stages.
- **Fn**: Denotes oligo-producing fructanases (exo-based) that predominantly produce products with a single DP at initial stages. The value of “n” may extend to DP8 or potentially even higher.
- **Fn,n+1**: Represents oligo-producing fructanases (exo-based) that yield a mixture of products ranging from DPn to DPn+1.
- **F1**: Refers to fructanases (exo-based) that prominently produce Fru from the very beginning of the reaction.

The same design can be applied to glucanases:

- **Gr**: Refers to random glucanases (endo-based), which do not produce significant Glc or glucan disaccharides at initial stages. Instead, they generate a very broad DP spectrum at intermediate stages.
- **Gn**: Denotes oligo-producing glucanases (exo-based) that predominantly produce products of a single DP at initial stages. The value of “n” may extend to DP8 or potentially even higher.
- **Gn,n+1**: Represents oligo-producing glucanases (exo-based) that yield a mixture of products ranging from DPn to DPn+1.
- **G1**: Refers to glucanases (exo-based) that prominently produce Glc from the very beginning of the reaction.

## 4. Good Practices

To reduce confusion about whether an enzyme operates in an exo- or endo-acting manner, it is essential to adhere to established “good practices”. The key guidelines outlined below will help ensure effective and accurate enzymatic studies. After determining the pH and temperature (T) optimum, the next important step in enzymatic studies is selecting the appropriate substrate (or array of candidate substrates). Ensuring substrate purity is crucial; it must be free from low-molecular-weight impurities that could interfere with the enzyme’s activity measurements (too-high background values, putative inhibitory effects). Such impurities should be removed (or at least significantly reduced) through extended dialysis or repeated alcohol or acetone precipitation. The lower the DP of the substrate, especially those with a polydisperse nature, the more challenging it becomes to capture the crucial “initial reaction conditions” window.

Ideally, the substrate should consist of at least 50 units, with 100 or more being preferable, to more effectively distinguish between the initial, intermediate, and terminal stages of enzymatic activity. For example, microbial levans, which have a very high DP (thousands of fructosyl units) and are readily soluble [13], serve as excellent substrates for discriminating between microbial endo- and exo-levanase enzyme degradation due to their extensive chain length.

However, these substrates are partially branched, leading to more complex degradation profiles and branched oligosaccharides that cannot be identified with the available sugar standards. Therefore, considering reaction products from more strictly linear plant-derived fructan substrates may help to interpret the generated product profiles [27,33].

In preliminary tests, it is recommended to use 2–3 different protein concentrations to capture the crucial “initial reaction conditions” window within the first 5–10 min of the enzymatic reaction. It is also recommended to perform preliminary enzyme stability studies to guarantee that the enzyme remains active during a certain timeframe, which is required to cover the full dynamics of the product spectra. To evaluate the enzyme’s catalytic efficiency and substrate affinity, it is important to determine its kinetic parameters such as the Michaelis–Menten constant (Km) and the maximum reaction rate (Vmax). Finally, confirming the enzyme’s molecular weight using Size Exclusion Chromatography (SEC) and Sodium Dodecyl Sulfate–Polyacrylamide Gel Electrophoresis (SDS-PAGE) is important to ensure it matches the expected size.

For F1/G1 enzymes, Fru or Glc production should be observed immediately, against a zero-time background free of Fru and Glc contamination. Fn/Gn enzymes are characterized by the immediate production of oligosaccharides without any background activity. For Fr/Gr enzymes, an initial high reducing capacity should be evident via the 3,5-dinitrosalicylic acid (DNS) method, with no immediate production of F1/G1 or Fn/Gn. A combination of techniques such as TLC, the DNS method, and HPAEC-IPAD offers the best guarantee of obtaining the critical initial product profile. Additionally, measurements should be taken at intermediate and endpoint stages to monitor the progression of enzyme activity. For F1/G1 enzymes, an increase in Fru and Glc production is expected. For Fn/Gn enzymes, a steady increase in oligosaccharide production with no polydisperse range of products should be observed. For Fr/Gr enzymes, a wide polydisperse product profile with a variety of products is anticipated at intermediate stages, while Fn,n+1/Gn,n+1-type enzymes are expected to show a few peaks from initial stages. HPAEC-IPAD may be required to fully discriminate between the two latter categories, accompanied by the use of appropriate sugar standards, if available.

## 5. Conclusions and Future Perspectives

How many true Fr/Gr-type enzymes exist in nature? It is likely that many previously characterized “endo” enzymes are not genuinely Fr/Gr type but rather Fn/Gn or Fn,n+1/Gn,n+1 types. This suggests the need for a reassessment of previously reported enzymes to ensure their accurate classification. To achieve this, it is crucial to follow the “good practice” methodology proposed in this study, which promotes consistency in enzyme classification across the field. Establishing a standardized approach will not only validate past findings but also ensure the accurate categorization of future discoveries.

As previously mentioned, the choice of substrates is critical for distinguishing between the proposed enzyme classes. When focusing on fructans, it is well known that the DP of plant-derived fructan substrates varies significantly across species. For example, chicory fructans typically have a low DP, ranging from 10 to 15, whereas inulins from *Echinops ritro* and *Viguiera discolor* and levans from *Dactylis glomerata* can exhibit DPs as high as 300 [34]. While low-DP plant fructans are unsuitable for time-dependent studies that allow proper classification, high-DP plant substrates are ideal for this purpose.

However, in general, the usability of high-DP carbohydrate polymers depends on their solubility. Another advantage is that plant-derived fructans are generally linear and lack side branches, unlike branched bacterial levans, which can contain 1000 to even 10,000 fructosyl units [13].

As previously discussed, the presence of side branches leads to a more complex degradation profile, producing branched oligosaccharides that cannot be accurately identified with the current sugar standards. To classify the candidate enzyme effectively, it is recommended to first test it on the high-DP plant-derived fructans mentioned above, which have a linear structure, and monitor the initial product formation. Alongside this, the enzyme can be tested on microbial-derived fructans to determine whether the product profile remains consistent with that observed for plant-derived fructans.

A relevant discussion on the hypothesis that many “endo” fructanase enzymes may actually be Fn- or Fn,n+1-type stems from previous work [35], which examined the “endo-inulinase” activity of the filamentous fungus *Aspergillus niger*. During its initial reactions with purified GF18 inulin, the enzyme primarily produced F3 and F4, similar to the endo-inulinase isolated from *Aspergillus ficuum* [22,30]. The authors collected the first sampling from the reaction mixture after 1 min, assuming it captured the true initial reaction products. According to our novel nomenclature system, this oligo-producing fructanase should be reclassified as F3,4, as the HPAEC-IPAD profile reveals a mixture of F3 and F4 products rather than a single DP product. This reclassification more accurately reflects the enzyme’s true product profile. To gain further certainty, the experiment should be repeated at lowered enzymatic activities, along with high-DP inulin from plant sources, as previously discussed. It would also be interesting to investigate whether this fungal enzyme can degrade bacterial inulin (e.g., derived from *Lactobacillus* or *Archaea* inulin) [36,37]. An intriguing question arising from this observation is whether true Fr-type enzymes exist at all within the fungal kingdom.

The proposed classification may need to be expanded in the future. One hypothesis suggests that some enzymes may switch between endo- and exo-activity during the course of the enzymatic reaction, starting with true endo-activity and gradually transitioning to exo-activity as the reaction progresses. Such a switching mechanism could be energy-efficient, as it would require only one enzyme instead of two. As the substrate is progressively hydrolyzed, smaller fragments are generated, increasing the likelihood of Fn-type hydrolysis at the non-sucrosyl, non-reducing end of GFn-type fructans. It can be hypothesized that such a switch may also be actively regulated. As the reaction progresses and small products accumulate, shorter DP products may bind to an allosteric site of the enzyme, triggering a conformational change that shifts the enzyme’s activity from Fr to Fn, or even F1. Further research, including molecular dynamics and docking studies focusing on substrate subsites, could explore the feasibility of such mechanisms. In Fn- and Fn,n+1-type enzymes, the minus subsites play a key role in stabilizing the catalytic complex. In contrast, Fr enzymes likely bind at random sites along high-molecular-weight polymers, enabling random cleavage events. These mechanistic insights may eventually lead to the design of mutants that switch the activity from endo- (Fr) to exo-type (Fn; Fn,n+1). Similar approaches could also be applied to glucan-degrading enzymes.

In conclusion, the proposed nomenclature system not only addresses current inconsistencies but also establishes a powerful and universal framework that can be applied to study potential fructanases and glucanases derived from various animal genomes. These were most likely acquired through horizontal gene transfer from bacterial sources [38]. Given this, it is essential to evaluate newly discovered animal-derived enzymes using our proposed nomenclature system to ensure accurate classification before investigating their complex physiological roles.

Furthermore, our novel classification system may be extended to a wide array of other endo- and exo-carbohydrate-hydrolyzing enzymes, such as arabinanases and chitinases, further enhancing clarity and precision in future enzyme classifications. Focusing on future applications with fructanases, it is evident that intensified research on endo–exo mechanisms and the new classification system will lead to the identification of novel, genuine Fr-type enzymes. This, in turn, will enhance our understanding of the exact retention times of IOS and LOS on HPAEC-IPAD, paving the way for their commercialization to serve research communities. Such advancements will further fuel investigations into the prebiotic effects of IOS and LOS, which are already well recognized for their beneficial physiological effects on human health, further establishing them as essential ingredients in the functional food industry [4,39]. In addition to their health benefits, IOS and LOS also support the growth of beneficial microbial communities in fermented foods such as natto, kombucha, and kimchi, which in turn offer significant health benefits and positively influence gut microbiota [39]. Beyond their prebiotic effects, recent studies suggest that IOS and LOS may also function as signaling molecules [40], potentially playing a role in bacterial quorum sensing (QS). QS is a process in which signaling molecules, or autoinducers, accumulate as the population increases, ultimately triggering coordinated behaviors such as motility, virulence, and biofilm formation [41]. QS is thought to be central in shaping beneficial bacterial communities during fermentation, within the gut, and in the plant rhizosphere. IOS and LOS are also emerging as promising priming compounds for plant protection [42,43], offering potential alternatives to traditional agrochemicals. These insights point to exciting prospects for the future, indicating substantial advancements and potential breakthroughs on the horizon.

## Data Availability

Not applicable.
